# A first-in-human phase I study to determine the maximum tolerated dose of the oral Src/ABL inhibitor AZD0424

**DOI:** 10.1038/bjc.2017.484

**Published:** 2018-02-13

**Authors:** Victoria K Woodcock, Sally Clive, Richard H Wilson, Vicky M Coyle, Michael R L Stratford, Lisa K Folkes, Richard Eastell, Claire Barton, Paul Jones, Shamim Kazmi-Stokes, Helen Turner, Sarah Halford, Adrian L Harris, Mark R Middleton

**Affiliations:** 1 University of Oxford Department of Oncology, Churchill Hospital, Old Road, Oxford OX3 7LJ, UK; 2 Edinburgh Cancer Centre, Western General Hospital, Edinburgh EH4 2XU, UK; 3 Centre for Cancer Research and Cell Biology, Queen's University Belfast, Lisburn Road, Belfast BT9 7AE, Northern Ireland, UK; 4 Oxford Institute for Radiation Oncology, Department of Oncology, University of Oxford, Old Road Campus Research Building, Roosevelt Drive, Oxford OX3 7DQ, UK; 5 Academic Unit of Bone Metabolism, University of Sheffield, Sheffield S10 2TN, UK; 6 Cancer Research UK Centre for Drug Development, Cancer Research UK, Angel Building, 407 St. John Street, London EC1V 4AD, UK; 7 National Institute for Health Research Oxford Biomedical Research Centre, Oxford OX3 7LE, UK

**Keywords:** AZD0424, Src, ABL, Phase I, targeted therapy

## Abstract

**Background:**

Src is involved in cancer invasion and metastasis. AZD0424, an oral inhibitor of Src and ABL1, has shown evidence of anti-tumour activity in pre-clinical studies.

**Methods:**

A phase Ia, dose escalation study was performed to assess the safety of continuous oral dosing with AZD0424 in advanced solid tumours. Secondary objectives included investigation of AZD0424 pharmacokinetics, effect on Src activity using markers of bone turnover, and anti-tumour activity.

**Results:**

41 patients were treated; 34 received AZD0424 once-daily at doses ranging from 5 mg to 150 mg, and 7 received 40 mg bi-daily 41.5% of patients experienced at least one AZD0424-related adverse event that was Grade 3–5 in severity, with patients treated at doses above 60 mg per day experiencing multiple treatment-related toxicities. The most commonly observed AZD0424-related adverse events were nausea, fatigue, anorexia and alopecia. *C*_max_ and AUC increased linearly with dose and the mean±standard deviation *t*_1/2_ was 8.4±2.8 h. Clear evidence of Src target inhibition was seen at doses ⩾20 mg per day. No responses were observed and 7 patients (17.1%) achieved stable disease lasting 6 weeks or more.

**Conclusions:**

AZD0424 displayed no evidence of efficacy as monotherapy despite a clear pharmacodynamic effect. Further evaluation of AZD0424 monotherapy in patients with solid tumours is not recommended.

AZD0424 is an inhibitor of the proto-oncogenic non-receptor tyrosine kinases Src and ABL1 which have been found to be dysregulated in cancer. Src is expressed at low levels in the majority of cell types and is implicated in pathways regulating bone metabolism, proliferation, survival, migration and angiogenesis (Aleshin and Finn, 2010). It is known to play a role in the regulation of cell invasion and metastasis in cancer, and elevated Src expression and activity is seen in several human tumour types including carcinomas of the breast (Egan *et al*, 1999), lung (Mazurenko *et al*, 1992) and colon (Bolen *et al*, 1987). In addition, Src expression has been correlated with advanced malignancy and poor prognosis in a variety of cancers (Wheeler *et al*, 2009a) including colorectal carcinoma (Aligayer *et al*, 2002) and osteosarcoma (Hu *et al*, 2015). There is also evidence that upregulation of Src expression and/or signalling is associated with resistance to many classes of anti-cancer drugs (Mayer and Krop, 2010) including those targeted against HER-2 (Peiro *et al*, 2014) and EGFR (Wheeler *et al*, 2009b), and that combination of Src inhibitors with such agents may reverse or prevent the occurrence of such resistance (Zhang *et al*, 2011). While more commonly known for its role in haematological malignancies, ABL1 kinase has also been identified as a potential target with relevance in solid tumours. For example, ABL kinases are constitutively activated in invasive breast cancer cell lines, downstream of deregulated ErbB receptors and Src kinases (Srinivasan and Plattner, 2006). It has also been shown that activation of ABL kinases can promote breast cancer cell invasion, and treatment of cells with the ABL1 kinase inhibitor, imatinib, markedly inhibits cell motility.

Accordingly, Src and ABL1 are potentially attractive targets for therapeutic intervention, and this has led to the clinical development of Src inhibitors such as saracatinib (Baselga *et al*, 2010), and dual Src/ABL1 inhibitors, including dasatinib (Yu *et al*, 2009) and bosutinib (Daud *et al*, 2011). While there has been efficacy in haematological malignancies, only occasional responses have been seen with these agents as monotherapy in solid tumours. Combination studies have also proven largely disappointing with no significant improvements in progression-free survival or overall survival seen in placebo-controlled trials of paclitaxel with saracatinib in platinum-resistant ovarian cancer (McNeish *et al*, 2014), or cediranib plus saracatinib in relapsed metastatic renal cell cancer (Powles *et al*, 2016). A large phase III trial of dasatinib plus docetaxel and prednisolone in patients with prostate cancer was also negative (Araujo *et al*, 2013) as was a smaller phase II study of cediranib alone *vs* cediranib in combination with dasatinib in docetaxel-resistant, castration-resistant prostate cancer (Spreafico *et al*, 2014). A lack of biomarkers, such as level of expression or activation of Src, to identify patients most likely to respond to therapy and contradictory effects of non-selective inhibitors upon different members of Src family kinases (SFKs), have been identified as potential issues underlying this lack of efficacy (Elias and Ditzel, 2015).

AZD0424 is an orally available, potent (IC_50_ approximately 4 nM) inhibitor of the Src and ABL1 kinases, with additional activity against other SFK members including Yes and Lck (Cancer Research UK Centre for Drug Development, 2015). Its chemical structure is displayed in Figure 1. AZD0424 exhibits considerable selectivity for Src-Abl1 kinases *vs* VEGFR-2 (>933-fold) and C-terminal Src tyrosine kinase (CSK) (>448-fold), a negative regular of Src kinase. While both dasatinib and bosutinib potently inhibit Src-Abl kinases (Davis *et al*, 2011), they have a relatively low selectivity for SFKs over CSK (approximately 5-fold and 30-fold respectively). An extensive kinase profile of Saracatinib has not been published; however, the available data suggests it has a similar prolife to that of AZD0424 (although the latter compound is somewhat more potent at inhibiting ABL1) (Green *et al*, 2009).

*In vitro*, AZD0424 demonstrated potent inhibition of proliferation of mouse fibroblasts engineered to over-express activated Src kinase (c-SRC 3T3) and also inhibited proliferation of growth factor stimulated human umbilical vein endothelial cell cultures. However, inhibition of proliferation in a diverse panel of non-engineered human tumour cell lines was generally poor. In contrast, AZD0424 produced potent inhibition of migration of human tumour cells, with evidence of inhibition of phosphorylation of the Src kinase substrate paxillin, suggesting anti-tumour effects may be due to inhibition of Src-mediated adhesion and motility signalling pathways. *In vivo*, AZD0424 produced moderate anti-tumour growth effects in rats bearing human Calu-6 lung tumour xenografts while profound, dose-dependent inhibition of tumour growth was seen in immunocompromised rats bearing subcutaneously growing c-Src 3T mouse xenografts. In pre-clinical studies, AZD0424 produced toxic effects in the gastrointestinal, haematopoietic and lymphoid systems, with gastrointestinal toxicity being dose limiting in both rat and dog. Hypotension and reflex tachycardia were also observed in both species. Bone turnover was found to be reduced with resultant increases observed in trabecular and cortical bone.

Based on the pre-clinical anti-tumour activity and acceptable toxicity profile, a first-in-man, phase Ia dose escalation study of AZD0424 was performed. The primary objective was to determine a recommended dose for AZD0424 as a single agent, by establishing the maximum tolerated dose (MTD) and assessing the safety profile of AZD0424 when given orally. The secondary objectives were to investigate the PK of AZD0424, its effect on markers of bone turnover (as proof of Src inhibition and achievement of a biologically active dose), and to explore possible anti-tumour activity in patients with advanced solid tumours.

## Patients and methods

### Patient eligibility

Patients ⩾18 years of age with histologically or cytologically proven solid tumours refractory to conventional treatment, or for which no suitable conventional therapy existed at the time, were eligible for the study. Other inclusion criteria included a life expectancy of at least 12 weeks, World Health Organisation (WHO) performance status of 0–2 and the following haematological and biochemical indices: haemoglobin ⩾9.0 g dl^−1^; absolute neutrophil count (ANC) ⩾1.5 × 10^9^ l^−1^; platelet count ⩾100 × 10^9^ l^−1^; serum bilirubin ⩽1.5 × upper limit of normal (ULN); alanine amino-transferase (ALT) and aspartate aminotransferase (AST) ⩽2.5 × ULN and calculated creatinine clearance ⩾50 ml min^−1^. All patients gave written informed consent in accordance with institutional guidelines before study treatment.

Patients were excluded from the study if they had symptomatic brain metastases, current significant or recent prior history of cardiac disease, QT interval prolongation (>480 ms, corrected for heart rate), hypotension (defined as systolic blood pressure <90 mm Hg) or other relevant clinical conditions such as active infection. Radiotherapy (except for palliative reasons), endocrine therapy, immunotherapy, chemotherapy or other investigational agents were not permitted during the 4 weeks before treatment with AZD0424 (6 weeks for nitrosoureas and mitomycin-C).

### Study design

CRUKD/07/61 was an open-label, first-in-human, phase I study. The starting dose of AZD0424 was 5 mg orally once daily (o.d.), based on pre-clinical studies which suggested a minimum anticipated biological effect level (MABEL) in a 70 kg human of approximately 5 mg. This dose was also less than one tenth of the maximum tolerated dose in rat scaled to man (17 mg). Treatment cycles consisted of 28 days of continuous AZD0424 administration, and treatment was to be continued until disease progression, patient withdrawal or unacceptable toxicity.

Patients were recruited initially into single patient cohorts until Grade 2 toxicity that was probably or possibly related to AZD0424 was observed in Cycle 1. Once Grade 2 drug-related toxicity was observed, that cohort was to be expanded to three patients and a ‘3+3’ dose escalation scheme initiated for the current and subsequent cohorts. Escalation of 100% of the dose between cohorts was permitted until a dose of 40 mg was reached, after which dose increments of up to 50% were allowed.

Dose-limiting toxicities (DLTs) were defined as adverse events (AEs) that were highly probably or probably related to AZD0424 during the first cycle of treatment including: Grade 4 neutropenia ⩾5 days; febrile neutropenia with ANC<1.0 × 10^9^ l^−1^; infection (documented clinically or microbiologically) with Grade 3 or 4 neutropenia (ANC<1.0 × 10^9^ l^−1^); Grade 2 diarrhoea for more than 7 days despite optimal treatment with anti-diarrhoeals; Grade 4 thrombocytopenia for ⩾5 days or associated with active bleeding or requiring platelet transfusion; any other Grade 3 or 4 non-haematological toxicity excluding Grade 3 nausea, Grade 3 or 4 diarrhoea or vomiting in patients who had not received optimal treatment, Grade 3 fatigue (unless there was an increase by at least two grades from baseline) or transient asymptomatic Grade 3 biochemical abnormalities. Later in the trial (from June 2015 during recruitment to the 40 mg bi-daily (b.d.) cohort), treatment-related AEs of any grade which together prevented administration of more than 25% of planned doses of AZD0424 during Cycle 1 were also considered dose-limiting. The MTD was defined as the dose below the dose level in which two out of up to six patients in a cohort experienced a DLT in the first cycle of AZD0424.

The study was conducted in accordance with the Declaration of Helsinki and the International Conference on Harmonisation of Good Clinical Practice Guidelines and approved by relevant regulatory and independent ethics committees.

### Patient evaluation

All patients receiving at least one dose of AZD0424 were evaluable for safety and efficacy analyses. Safety and tolerability of AZD0424 were assessed according to NCI CTCAE version 4.02. Patients who were withdrawn from treatment, or received less than 75% of the planned doses of AZD0424 during the first cycle, for reasons other than toxicity were not evaluable for dose review decisions and could be replaced. Disease response was assessed according to RECIST 1.1. For stable disease (SD), follow-up measurements had to meet the SD criteria at least once at least 6 weeks after the first dose of AZD0424.

### Dose modifications and delays

Treatment interruptions of up to 2 weeks were permitted for toxicities of Grade 2 or higher related to AZD0424 to allow resolution of toxicity to ⩽Grade 1 or to meet the eligibility criteria. Dose reductions to the previous dose level were permitted to manage toxicities.

### Pharmacokinetic analysis

Patients were fasted for 3 h prior to administration of AZD0424. Blood samples for analysis of plasma levels of AZD0424 were taken at the following time points: Cycle 1: Pre-dose and at 0.5, 1, 2, 4, 6 and 10 h post-dose on Day 1, then at 24 and 48 h and pre-dose on Days 8, 15 and 22; Cycle 2 (Day 29): Pre-dose and at 0.5, 1, 2, 4, 6, 10, 24 and 48 h. For patients receiving twice-daily AZD0424, the timing of samples was in relation to the morning dose of the drug. Plasma was isolated from centrifuged blood samples and stored at −80 °C prior to quantification of AZD0424 by a validated high performance liquid chromatography method with mass spectrometric detection. Plasma concentration/time data were analysed using non-compartmental methods.

### Pharmacodynamic analysis

Src inhibition is known to reduce bone resorption, resulting in a decrease in urinary N-terminal cross-linking telopeptides of type I collagen (NTX) and serum C-terminal cross-linking telopeptides of type I collagen (CTX) (Hannon *et al*, 2012). Measurements of urinary NTX and serum CTX levels were performed before the first administration of AZD0424 and then weekly, at the same time of day, for up to 6 weeks following first administration as markers of Src inhibition. Samples were analysed at the Mellanby Centre for Bone Research in Sheffield using the Cobas e411 automated immunoassay (Roche Diagnostics, Mannheim, Germany) for serum CTX (ng ml^−1^), and the Ortho Clinical Diagnostics (High Wycombe, UK) automated immunoassay for urine NTX. Urinary NTX measurements were reported as a ratio to urinary creatinine (nmol bone collagen equivalent (BCE)/mmol creatinine). Tumour biopsies for measurement of tumour biomarkers (p-Src, Ki67, p-PAX, and p-FAK) by immunohistochemical staining were optional.

## Results

### Patient characteristics

A total of 43 patients were enrolled in the study between 25 October 2012 and 18 November 2015 at three UK Experimental Cancer Medicine Centres, of whom 41 received AZD0424. Baseline characteristics are summarised in Table 1.

Nineteen dosed patients (46.3%) had colorectal cancer while the remainder had a range of other solid tumours. All 41 patients had RECIST measurable disease at baseline and 90% of patients had more than one site of disease involvement. All patients enrolled onto the study had received prior chemotherapy and most were heavily pre-treated.

### Dose escalation and MTD

The dose levels explored, number of patients treated and DLTs are summarised in Table 2. Expansion to three-patient cohorts occurred at 20 mg per day triggered by Grade 2 hypophosphataemia possibly related to AZD0424. Further escalation continued (see Supplementary Information) until two patients experienced DLTs (Grade 3 maculopapular rash, and Grade 3 fatigue, anorexia and maculopapular rash) in the 150 mg cohort leading to this cohort being declared non-tolerated. With one DLT (Grade 3 diarrhoea and melaena) at 120 mg o.d. in an expanded patient cohort, this was declared the MTD. Given that considerable drug-related toxicity was seen at this level, twice daily dosing of AZD0424 at 40 mg b.d. was explored, but two DLTs occurred at 40 mg b.d. and this was less well tolerated than 80 mg o.d., so b.d. dosing was not investigated further.

### AZD0424 administration, safety and tolerability

A total of 82 cycles of AZD0424 treatment were administered with patients on treatment for a median of 45 days (range 4–182 days). Fifteen patients were withdrawn from the study prior to completing one cycle of treatment. Six patients were withdrawn due to AEs or SAEs, four due to disease progression and five due to other reasons (patient request=3; interruption of treatment >2 weeks unrelated to AZD0424=1; and combination of progressive disease (PD) and several AEs/SAEs=1). Twenty-six patients (63.4%) had dose delays and/or alterations during the study; however, overall treatment compliance was satisfactory with 68% of patients receiving ⩾75% of their planned treatment in Cycle 1 and 39% of these receiving 100% of their planned treatment.

Of the 41 patients treated, 38 (92.7%) experienced at least one AE that was considered possibly, probably or highly probably related to AZD0424, and 17 patients (41.5%) had at least one AZD0424-related event that was Grade 3–5 in severity. The mean number of treatment-related AE episodes per patient and the number of treatment-related Grade 3, 4 or 5 AEs increased markedly at doses above 60 mg per day (Table 2). Nausea, fatigue, anorexia and alopecia were the most commonly reported treatment-related AEs, each occurring in >40% of patients (Table 3). Hypophosphataemia was the most commonly observed laboratory abnormality of any cause seen during the study, with changes in calcium levels also apparent but less marked. A decline in lymphocyte count was observed in 28/41 patients (68.3%) but was not associated with an apparent increase in infections.

Four patients died within 4 weeks of the last AZD0424 administration, all of whom had withdrawn from the study before completion of one cycle of AZD0424. Two of these deaths were due to disease progression, and two were due to pneumonia. One death due to pneumonia was considered possibly related to AZD0424.

### Pharmacokinetics

**Figure 2:**
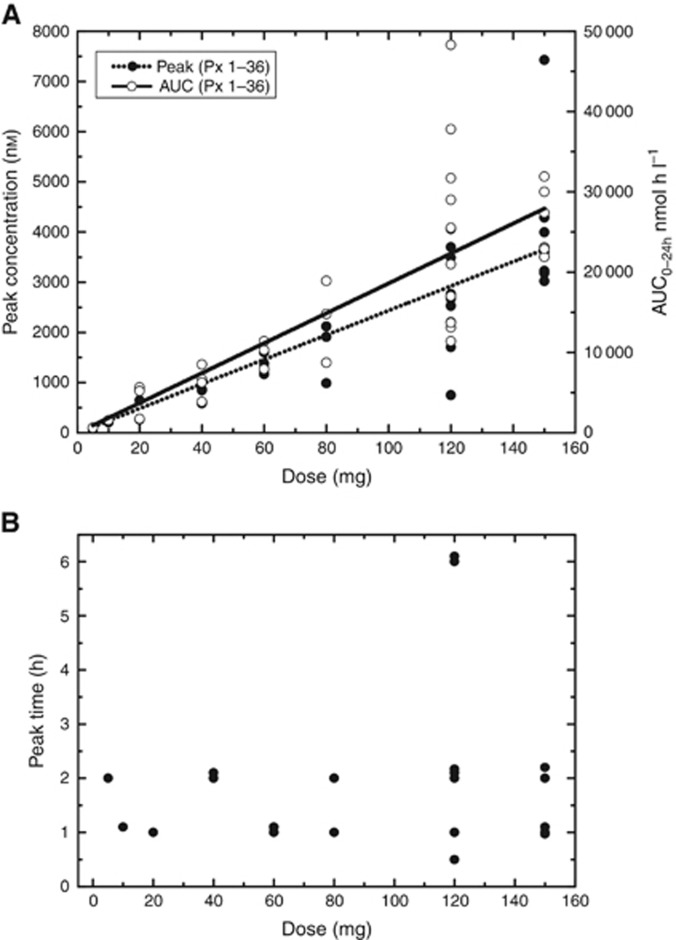
**Pharmacokinetic data for once daily dosing of AZD0424.** (**A**) Relationship between AZD0424 dose and peak concentration (*C*_max_) and AUC_(0–24 h)_ for Cycle 1. (**B**) Relationship between AZD0424 dose and time to reach peak concentration (*T*_max_) for Cycle 1.

When given o.d., AZD0424 was cleared from plasma with a mean terminal half-life (*t*_1/2_) of 8.4±2.8 h (mean±s.d.) (Supplementary Table 1). Linear, dose-dependent increases in maximum concentration (*C*_max_) and the area under the concentration-time curve from 0–24 h (AUC_(0-24 h)_) were seen (Figure 2A), while the time to reach maximum concentration (*T*_max_) was independent of dose and under 2 h in almost all patients (Figure 2B). Both *C*_max_ and AUC_(0-24 h)_ were much higher for AZD0424 than saracatinib at comparable doses (Baselga *et al*, 2010) (Supplementary Figure 1). *t*_1/2_ and AUC could not be accurately determined for the b.d. cohort as the sampling schedule did not capture sufficient data before the second daily dose.

Despite a greater number of AEs experienced by individual patients at higher dose levels, no clear relationships could be discerned between any PK parameter and individual AEs, with the possible exception of hypophosphataemia where Grade 2 or 3 hypophosphataemia appeared to be more frequent in patients with minimum concentration levels (*C*_min_)⩾200 nM (data not shown).

### Pharmacodynamics

**Figure 3:**
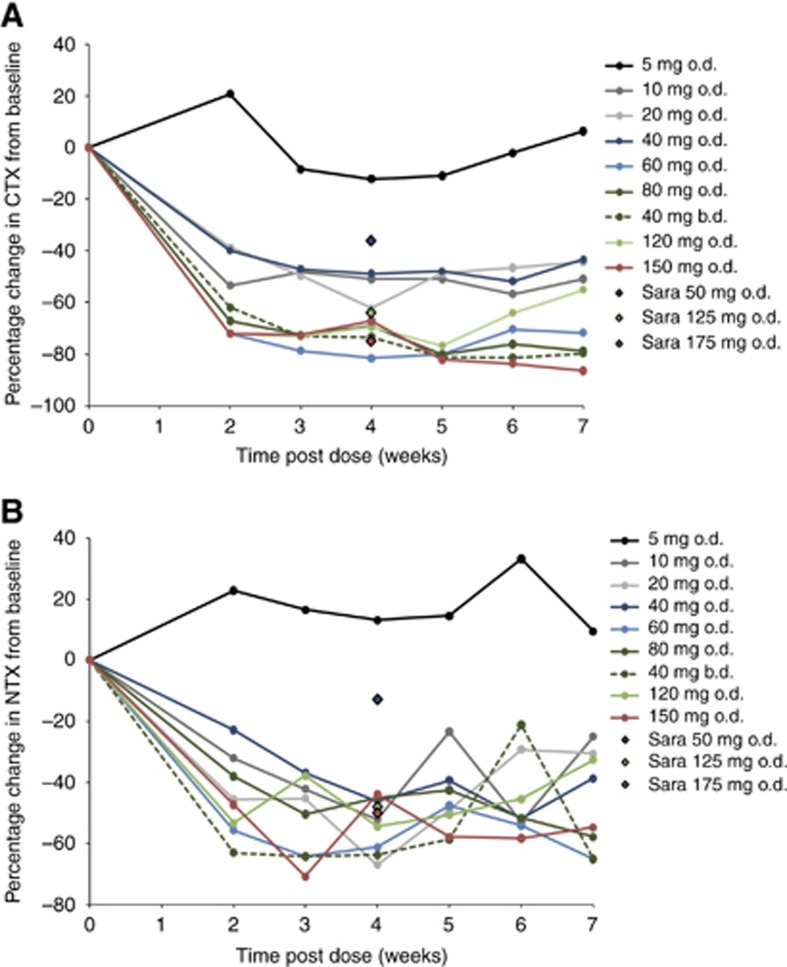
**(A) CTX levels over time, expressed as a percentage of baseline levels.** (**B**) NTX levels over time, expressed as a percentage of baseline levels. Published data for saracatinib are also shown (black edged diamonds labelled ‘Sara’) (Hannon *et al*, 2012). b.d.=bi-daily; o.d.=once-daily.

No significant post-administration changes in CTX or NTX level were observed in the single patient who received AZD0424 5 mg. In all other cohorts, a fall relative to baseline values in both CTX and NTX was observed by week 2 and maintained to 7 weeks (Figure 3). Greater percentage reductions in CTX and NTX levels were seen at doses of more than 10 mg per day of AZD0424 than that achieved with 50 mg per day of saracatinib and for NTX, similar to that achieved with 125 or 175 mg per day of saracatinib (Hannon *et al*, 2012) (Figure 3). No patients consented to undergo a tumour biopsy during the study.

### Efficacy

No patients who received AZD0424 achieved a complete response or partial response. A total of seven patients (17.1%) had a best response of SD (lasting at least 6 weeks), of whom five achieved SD lasting >12 weeks. Twenty-two patients had a best response of PD and 12 patients had no tumour response recorded. There were no obvious shared characteristics among the patients who experienced SD.

## Discussion

In this study AZD0424 was evaluated as monotherapy at total daily doses ranging from 5 mg to 150 mg in 41 patients with advanced solid tumours. PK data indicated rapid absorption and high bioavailability with dose-dependent, linear increases in *C*_max_ and AUC. Clear inhibition of bone turnover was achieved with AZD0424 doses of 20 mg per day or more, indicating that almost all the patients in the study received AZD0424 doses that achieved Src target inhibition. Both plasma drug concentrations and reductions in markers of bone turnover were considerably higher with AZD0424 than those seen in phase I trials of the closely related Src inhibitor saracatinib at similar doses.

The toxicity profile of AZD0424 was generally consistent with expectations based on non-clinical studies of AZD0424 and clinical data for other Src/ABL inhibitors. Gastrointestinal disorders were the most frequently observed AZD0424-related toxicities including nausea, anorexia and vomiting. A similar profile of toxicities was observed with saracatinib but the grade and frequency of these findings was lower than found with AZD0424 (Baselga *et al*, 2010). Hypophosphataemia was also common with AZD0424, with eight patients (19.5%) experiencing an AE of Grade 3 hypophosphataemia related to the drug. This is relatively high compared with most other saracatinib monotherapy studies where this finding was rare or absent (Baselga *et al*, 2010; Gucalp *et al*, 2011; Gangadhar *et al*, 2013), although a 50% rate of Grade 3 hypophosphataemia was reported in a phase II trial of this agent in colorectal cancer patients (Reddy *et al*, 2015). The investigators postulated that the presence of malignancy involving the gastrointestinal tract might have rendered colorectal patients particularly sensitive to this phenomenon; however, our study showed no apparent predisposition to hypophosphataemia with AZD0424 for colorectal cancer patients. Greater exposure was found with AZD0424 at a dose of 20 mg than observed with saracatinib at its recommended phase II dose (RP2D) of 175 mg (Baselga *et al*, 2010). As saracatinib and AZD0424 have a similar potency for Src inhibition, this greater exposure to AZD0424 may have led to a greater degree of Src inhibition and more extensive off-target kinase inhibition than found with the former agent. No clear correlations were seen between any individual AEs and PK parameters. This may be explained by a threshold effect once a certain degree of Src inhibition is achieved or by the lack of data points at lower doses.

Based on the DLTs and AEs observed during this study, the protocol-defined RP2D for AZD0424 would be 80 mg o.d.; however, in practice this may not be suitable for long-term dosing based on the burden of Grade 1 and 2 AEs. This phenomenon of cumulative low grade toxicity is increasingly recognised with the use of molecularly targeted agents and there are calls for DLT criteria to be adapted accordingly (Postel-Vinay *et al*, 2014), for example to include any drug-related toxicity that prevents administration of >75% of planned doses within the first cycle as a DLT, and for relative dose intensity to be factored into determination of the RP2D. Given that clear pharmacodynamic effects on bone turnover were seen at relatively low doses of AZD0424, the question also arises as to the appropriateness of using the traditional model of dose escalation to the MTD in phase I clinical trials of molecularly targeted agents. While for chemotherapeutic agents, greatest efficacy has historically been seen predominantly at higher doses close to the RP2D (Von Hoff and Turner, 1991), more recent data suggest that this does not necessarily hold true for molecularly targeted agents with patients treated at lower doses experiencing efficacy outcomes no worse than those treated at higher doses (Jain *et al*, 2010). Indeed, the importance and need to determine the MTD of novel agents in the era of biomarker-driven research is the subject of debate (Haines, 2008; Sleijfer and Wiemer, 2008). Given the potential for loss of kinase selectivity as doses increase, and that increased toxicity at higher doses of AZD0424 in this study significantly limited tolerability, it is possible that the minimal biologically effective dose might have been a more clinically relevant parameter than MTD.

Despite the pharmacodynamic evidence of Src target inhibition by AZD0424, there was no evidence of anti-tumour activity in this study. As no tumour biopsies were available, we were unable to analyse the Src status of the patients recruited to determine whether any patients had disease that might be expected to be responsive to Src inhibition. The rate of PD (22/41, 53.7%) was comparable to that seen in other published phase I studies (Horstmann *et al*, 2005; Italiano *et al*, 2008; Wong *et al*, 2016), which is typically around 50%. A significant number of patients left the study before the first disease assessment at 6 weeks. Seven patients (17.1%) had documented SD, a figure similar to that found in the phase I trial of saracatinib (Baselga *et al*, 2010). Concerns were raised about the rate of disease progression in two individual patients dosed at 40 mg b.d. and 150 mg o.d. One other patient (treated with 40 mg b.d.) experienced a rapid clinical decline that reversed on cessation of treatment. A detailed review of individual patient and overall trial data found no convincing evidence to suggest that AZD0424 was accelerating the rate of disease progression. It did, however, suggest that the cumulative lower grade toxicities experienced by patients taking AZD0424 at higher dose levels may have contributed to deteriorating clinical condition.

Based on the lack of efficacy seen in this study, further evaluation of AZD0424 monotherapy in patients with solid tumours is not recommended. Combination dosing with other agents might require further dose reduction of AZD0424 in order to be tolerable; however, emergent data from pre-clinical work have failed to show sufficient promising evidence of synergy for AZD0424 with other agents to warrant further investigation.

## Figures and Tables

**Figure 1:**
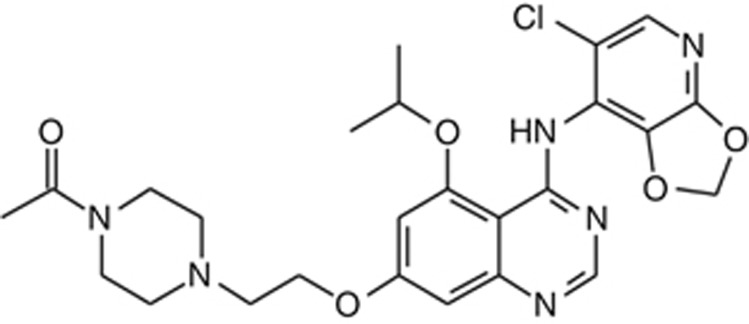
**Structural formula of AZD0424 (7-[2-(4-Acetylpiperazin-1-yl)ethoxy]-*N*-(6-chloro[1,3]dioxolo[4,5-b]pyridin-7-yl)-5-isopropoxyquinazolin-4-amine).**

**Table 1:** Summary of baseline demographic and disease characteristics (*N*=41 patients)

Age, median (range)	
Years	59 (38–76)
Gender, *n*(%)	
Male	22 (54)
Female	19 (46)
Performance status, *n*(%)	
0	15 (37)
1	25 (61)
2	1 (2)
Primary tumour type, *n*(%)	
Colorectal	19 (46)
Lung	3 (7)
Pancreas	2 (5)
Bile duct	2 (5)
Cervical	2 (5)
Breast	2 (5)
Anal	2 (5)
Other^a^	9 (22)
Disease sites at study entry, *n*(%)	
Liver metastases	27 (66)
Lung metastases	25 (61)
Lymph nodes	21 (51)
Peritoneal metastases	13 (32)
Soft tissue	7 (17)
Bone metastases	3 (7)
Other^b^	22 (54)
Prior treatment, *n*(%)	
Chemotherapy	41 (100)
No. regimens 1–3	24 (59)
4–6	15 (37)
⩾7	2 (5)
Surgery	33 (80)
Radiotherapy	17 (41)

a Lower oesophagus (1), SCC cheek (1), epithelioid mesothelioma (1), GIST (1), ocular melanoma (1), ovary (1), peritoneal carcinoma (1), pleura (1), unknown primary (1).

b Local recurrence (3), malignant pleural effusion (1), primary tumour (6), other metastases (12).

**Table 2:** Dose levels, dose limiting toxicities and AZD0424-related AE episodes

**Cohort**	**AZD0424 dose mg**	**Patients treated**	**Total cycles AZD0424 started**	**Patients evaluable**	**Patients experiencing DLT**	**Description of DLT**	**Related AE episodes per patient** **(mean (range))**	**Related Grade 3–5 AE episodes per patient** **(mean (range))**
1	5 mg o.d.	1	2	1	0		2.0 (2 to 2)	0
2	10 mg o.d.	1	7	1	0		5.0 (5 to 5)	0
3	20 mg o.d.	4	9	3	0		7.33 (6 to 9)	0
4	40 mg o.d.	4	13	3	0		4.50 (1 to 7)	1.0 (1 to 1)
5	60 mg o.d.	3	5	3	0		4.0 (4 to 4)	1.0 (1 to 1)
6	80 mg o.d.	3	5	3	0		9.50 (5 to 14)	0
7	120 mg o.d.	11	19	7	1	G3 diarrhoea and melaena	8.90 (3 to 19)	2.50 (1 to 4)
8	150 mg o.d.	7	11	5	2	G3 maculopapular rash G3 fatigue, anorexia and maculopapular rash	10.14 (1 to 22)	3.0 (1 to 8)
9	40 mg b.d.	7	11	6	2	G3 fatigue Completed <75% of Cycle 1 doses due to combination G2 nausea, fatigue, and anorexia	12.29 (5 to 17)	2.75 (2 to 4)

Abbreviations: AE=adverse event; b.d.=bi-daily; DLT=dose-limiting toxicity; o.d.=once-daily.

**Table 3:** Treatment-related AEs by NCI-CTCAE Grade v4.02

**AE (occurring in >10% of patients)**	**All grades patients** ***n*** **(%)**	**Grade 3–5 patients** ***n*** **(%)**
Nausea	27 (66)	2 (5)
Fatigue	21(51)	5 (12)
Anorexia	19 (46)	1 (2)
Alopecia	17 (41)	0
Hypophosphataemia	16 (39)	8 (20)
Dysgeusia	14 (34)	0
Vomiting	13 (32)	1 (2)
Diarrhoea	12 (29)	2 (5)
Lymphocyte count decreased	10 (24)	5 (12)
Maculopapular rash	9 (22)	2 (5)
Pruritis	7 (17)	0
Anaemia	7 (17)	3 (7)
Constipation	6 (15)	1 (2)
Headache	5 (12)	0
Aspartate aminotransferase increased	5 (12)	0

Abbreviation: AE=adverse event.

Other ⩾G3 treatment-related AEs occurring in any patient (*n*) – G3 neutrophil count decreased (1), G3 lower gastrointestinal haemorrhage (1), G3 melaena (1), G5 lung infection (1), G3 chest infection (1), G3 hyponatraemia (1), G3 acute kidney injury (1).
