# Supplementary material for: A first-in-human phase I study to determine the maximum tolerated dose of the oral Src/ABL inhibitor AZD0424
Source: Br J Cancer. 2018 Feb 13;118(6):770–6. doi: 10.1038/bjc.2017.484 (PMC5877436; doi:10.1038/bjc.2017.484)
Supplement: Supplementary Information [file bjc2017484x1.docx]

**Supplementary Information**

**Dose escalation scheme**

AZD0424 was administered orally on a daily basis at a starting dose of 5 mg, initially with single patient cohorts. After two dose doublings to 20 mg/day an AE of Grade 2 hypophosphataemia, considered to be possibly related to AZD0424, was observed. As this was the first AZD0424-related Grade 2 toxicity, as per protocol, the 20 mg cohort was expanded to a three-patient cohort and subsequent cohorts proceeded according to a ‘3+3’ design. On the basis of multiple low grade related AEs not meeting DLT criteria in the initial patients treated in the 120 mg cohort, the decision was taken to expand this cohort before escalating further. After five evaluable patients were treated without DLTs, the decision was taken to escalate by 25% to 150 mg in an attempt to determine the MTD. In the subsequent 150 mg cohort, two patients experienced DLTs (Grade 3 maculopapular rash, and Grade 3 fatigue, anorexia and maculopapular rash) leading to this cohort being declared non-tolerated. As five evaluable patients had previously been treated at 120 mg, this cohort was expanded and two further patients were treated at 120 mg o.d. With 1 DLT (Grade 3 diarrhoea and melaena) in this expanded cohort, 120 mg o.d. was declared the MTD. Given that considerable drug related toxicity was seen at this level, the decision was made to investigate the tolerability of twice daily dosing and a cohort of patients were treated at 40 mg b.d. Two DLTs were observed in six evaluable patients at this level, one patient experienced Grade 3 fatigue, the other missed > 25% of planned doses due to a combination of Grade 2 nausea, fatigue and anorexia. As a result, 40 mg b.d. was declared non-tolerated and further b.d. dosing regimens were not explored.

**Supplementary Table 1.** Average PK data for single-daily dose AZD0424

| Dose o.d mg | Cycle | AUC_(0-24h)_ nmol.h.L^-1^ (mean±SD) | t_½_ h (mean±SD) | C_max_ nM (mean±SD) | Time to C_max_ h (mean±SD) |
| --- | --- | --- | --- | --- | --- |
| 5 | 1 | 624 | 6.7 | 68.3 | 2.0 |
|  | 2 | 746 | 6.0 | 104 | 1.1 |
| 10 | 1 | 1570 | 6.3 | 210 | 1.1 |
|  | 2 | 1700 | 6.2 | 267 | 1.0 |
| 20 | 1 | 4193±2145 (n=3) | 6.8±2.6 (n=3) | 590±300 (n=3) | 1.0±0 (n=3) |
|  | 2 | 8550 | 12 | 640 | 1.0 |
| 40 | 1 | 6325±1904 (n=4) | 10.2±3.4 (n=4) | 787±134 (n=4) | 2.1±0.1 (n=4) |
|  | 2 | 6810±3463 (n=3) | 10.5±0.8 (n=3) | 715±380 (n=3) | 2.0±0 (n=3) |
| 60 | 1 | 9880±1768 (n=3) | 8.2±3.3 (n=3) | 1384±215 (n=3) | 1.0±0.1 (n=3) |
|  | 2 | 13350±2899 (n=2) | 11.1±5.5 (n=2) | 1628±706 (n=2) | 1.5±0.7 (n=2) |
| 80 | 1 | 14140±5122 (n=3) | 9.4±5.0 (n=3) | 1673±602 (n=3) | 1.3±0.6 (n=3) |
|  | 2 | 15457±7577 (n=6)* | 8.5±1.5 (n=6)* | 1673±735 (n=6)* | 2.0±2.1 (n=6)* |
| 120 | 1 | 24850±12027 (n=10) | 7.8±3.1 (n=10) | 2646±923 (n=11) | 2.5±1.9 (n=11) |
| 120 | 2 | 21900±4462 (n=4) | 8.6±3.8 (n=4) | 2573±576 (n=4) | 2.2±1.2 (n=4) |
| 150 | 1 | 25586±4147 (n=7) | 7.8±2.1 (n=7) | 4106±1536 (n=7) | 1.3±0.5 (n=7) |
| 150 | 2 | 32550±10112 (n=2) | 7.0±0.1 (n=2) | 4324±1272 (n=2) | 1.0±0 (n=2) |

* Includes data where dose was reduced to 80 mg in Cycle 2

**Supplementary Figure 1.** Comparison of the C_max_ and AUC_0-24h_ for AZD0424 at doses up to 40mg, and published data for saracatinib at 50 mg ([Baselga *et al*, 2010](#_ENREF_2)).
